# Interaction between Wnt/β-catenin and RAS-ERK pathways and an anti-cancer strategy via degradations of β-catenin and RAS by targeting the Wnt/β-catenin pathway

**DOI:** 10.1038/s41698-018-0049-y

**Published:** 2018-02-20

**Authors:** Woo-Jeong Jeong, Eun Ji Ro, Kang-Yell Choi

**Affiliations:** 1 Translational Research Center for Protein Function Control, Yonsei University, Seoul, Korea; 2 Department of Biotechnology, College of Life Science and Biotechnology, Yonsei University, Seoul, Korea

## Abstract

Aberrant activation of the Wnt/β-catenin and RAS-extracellular signal-regulated kinase (ERK) pathways play important roles in the tumorigenesis of many different types of cancer, most notably colorectal cancer (CRC). Genes for these two pathways, such as *adenomatous polyposis coli* (*APC*) and *KRAS* are frequently mutated in human CRC, and involved in the initiation and progression of the tumorigenesis, respectively. Moreover, recent studies revealed interaction of *APC* and *KRAS* mutations in the various stages of colorectal tumorigenesis and even in metastasis accompanying activation of the cancer stem cells (CSCs). A key event in the synergistic cooperation between Wnt/β-catenin and RAS-ERK pathways is a stabilization of both β-catenin and RAS especially mutant KRAS by *APC* loss, and pathological significance of this was indicated by correlation of increased β-catenin and RAS levels in human CRC where *APC* mutations occur as high as 90% of CRC patients. Together with the notion of the protein activity reduction by lowering its level, inhibition of both β-catenin and RAS especially by degradation could be a new ideal strategy for development of anti-cancer drugs for CRC. In this review, we will discuss interaction between the Wnt/β-catenin and RAS-ERK pathways in the colorectal tumorigenesis by providing the mechanism of RAS stabilization by aberrant activation of Wnt/β-catenin. We will also discuss our small molecular anti-cancer approach controlling CRC by induction of specific degradations of both β-catenin and RAS via targeting Wnt/β-catenin pathway especially for the KYA1797K, a small molecule specifically binding at the regulator of G-protein signaling (RGS)-domain of Axin.

## Introduction

Cancer is a complex disease involving multiple mutations of genes including those involved in the many different signaling pathways. However, the relationships between mutations of the genes involving different pathways are poorly understood. The Wnt/β-catenin and RAS-ERK pathways are major pathways involving human cancers, and abnormalities in these two pathways genes are well characterized, such as *APC* and *CTNNB1* (the gene encoding β-catenin) mutations in the Wnt/β-catenin pathway and *RAS*, *RAF*, and upstream epidermal growth factor receptor (EGFR) mutations in the RAS-ERK pathway have been known to play important roles in the tumorigenesis. In human colorectal cancer (CRC), series of gene mutations are involved in the tumorigenesis, and the accumulation of genetic and epigenetic alterations are involved in the tumorigenesis.^1,2^ The tumor suppressor APC functions as a “gatekeeper” and its gene mutations occur at the initiation stage of the colorectal tumorigenesis, and *KRAS* mutations occur at the early adenoma stage and play roles in the progression of the tumorigenesis.^2^ The *APC* and *KRAS* mutations frequently occurs simultaneously; however, interaction of these two gene mutations in the tumorigenesis is poorly understood. Over the past decade, significant progress has been made in understanding the mechanism of cross-talk between the Wnt/β-catenin and RAS-ERK pathways in cancer. *APC* and *KRas* or *CTNNB1* and *HRas* mutations resulted in the cooperatively progressed tumorigenesis in mouse genetic studies.^3–5^ In addition, the CRC patients with both *APC* and *KRAS* mutations reveled much severe cancer phenotypes compared with their single mutation.^2^ Furthermore, both *APC* and *KRAS* mutations resulted in the activation of cancer stem cells (CSCs) even progression to metastasis in the mouse xenograft model.^6^ Overall, the Wnt/β-catenin and RAS-ERK pathways interact in the various stages of the colorectal tumorigenesis and combined mutations of these two pathway genes are critical in the malignant transformation of CRC. In this review, we are going to discuss the current understanding for the roles of the cross-talk between the Wnt/β-catenin and RAS-ERK pathways in tumorigenesis especially for CRC, and also will discuss the mechanical base for the interaction between the two pathways highlighted by the stability regulation of RAS via the Wnt/β-catenin pathway. Finally, we will discuss our promising small molecular approach controlling CRC where both β-catenin and RAS especially mutant KRAS levels were increased by their stabilization by *APC* loss in human CRC.

## The Wnt/β-catenin signaling pathway

**Fig. 1:**
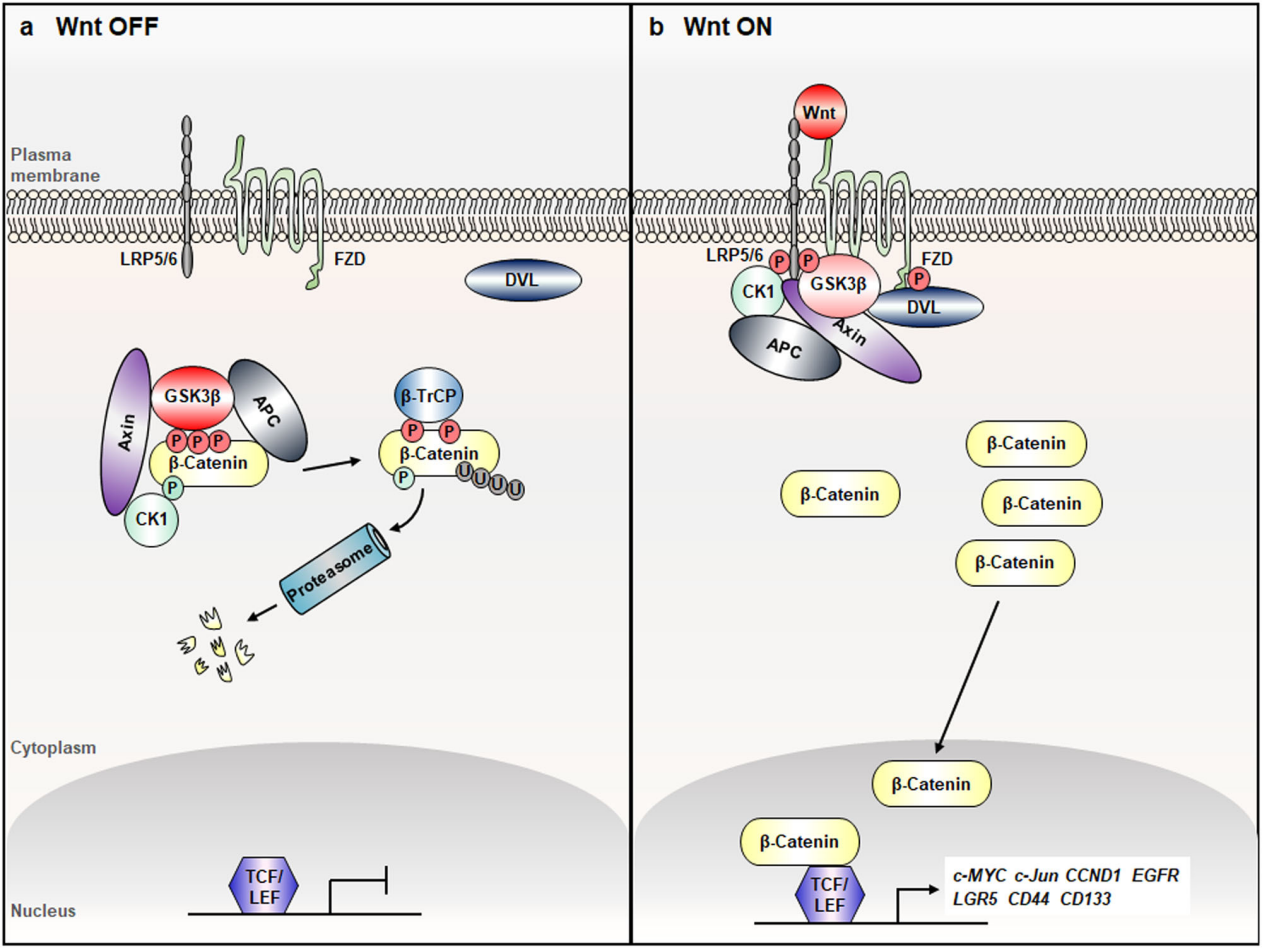
The canonical Wnt/β-catenin pathway. **a** In the absence of Wnt ligands, APC (adenomatous polyposis coli) and Axin are recruited into the “β-catenin destruction complex”. The phosphorylations by CK1α (casein kinase 1α) and GSK3β (glycogen synthase kinase 3β) recruit β‑TrCP E3 linker (β-transducin repeat-containing protein, an E3 ubiquitin ligase), and subsequently degrade β‑catenin via the proteasome. Low-cytoplasmic levels of β-catenin ensure in activation of TCF/LEF (T-cell factor/lymphoid enhancer factor) transcription factors and transcriptional repression of Wnt target genes. **b** In the accumulation of the extracellular Wnt ligands, the association of Axin with phosphorylated LRP5/6 (lipoprotein receptor-related protein 5/6) and recruitment of phosphorylated DVL (dishevelled) to FZD (frizzled) lead to the dissociation of the destruction complex. β-Catenin is stabilized, translocated into the nucleus, forms the complex with TCF or LEF, and subsequently activates the target genes. CCND1, cyclin D1; EGFR, epidermal growth factor receptor; LGR5, Leucine-rich repeat-containing G-protein coupled receptor 5; P, phosphorylation; U, ubiquitination

The Wnt/β-catenin signaling pathway has been conserved throughout evolution and is crucial during both normal embryonic development and throughout the life to maintain homeostasis of in virtually every tissue.^7^ Moreover, Wnt/β-catenin signaling has crucial roles in the daily processes of tissue maintenance and regeneration in the hair, skin and intestine, and so on.^8^ The Wnt/β-catenin pathway is initiated by Wnt family ligands, secreted lipoglycoproteins that act to the frizzled (FZD) family receptors.^9^ The key signaling molecule β-catenin determining the activation status of this pathway subjected to the stability regulation at the cytosol. In the resting status without Wnt stimuli, the cytoplasmic β-catenin form the ‘destruction complex’ composed with the casein kinase 1α (CK1α), glycogen synthase kinase 3β (GSK3β), APC, and Axin, and its level is maintained as low by the series of events including priming phosphorylation by CK1α at Ser45 and subsequently at Thr41, Ser37, and Ser33 by GSK3β,^9,10^ and recruitment of the β-TrCP E3 linker followed by degradation through the polyubiquitination-mediated proteasomal degradation.^11^ When the secreted Wnt ligands are accumulated, those interact to the FZD receptor together with the lipoprotein receptor-related protein (LRP)-5/6 co-receptors result in activation of dishevelled (DVL) protein (Fig. 1).^12^ The activated DVL is phosphorylated and translocate to the FZD receptor^13,14^ and dissociate the β-catenin destruction complex followed by its cytosolic accumulation and subsequent translocation into the nucleus. The nuclear accumulated β-catenin form complex with the T-cell transcription factor (TCF) or lymphoid enhancer factor (LEF), and activate expression of the target genes involving various pathophysiologies including the genes involving proliferation and transformation, such as *c-MYC*,* c-Jun*,* CCND1* (the gene encoding cyclin D1), *EGFR*,* CD44*, *CD133*, and *leucine-rich repeat-containing receptor 5* (*LGR5)* and so on (Fig. 1).^15–19^

## The RAS-ERK signaling pathway

**Fig. 2:**
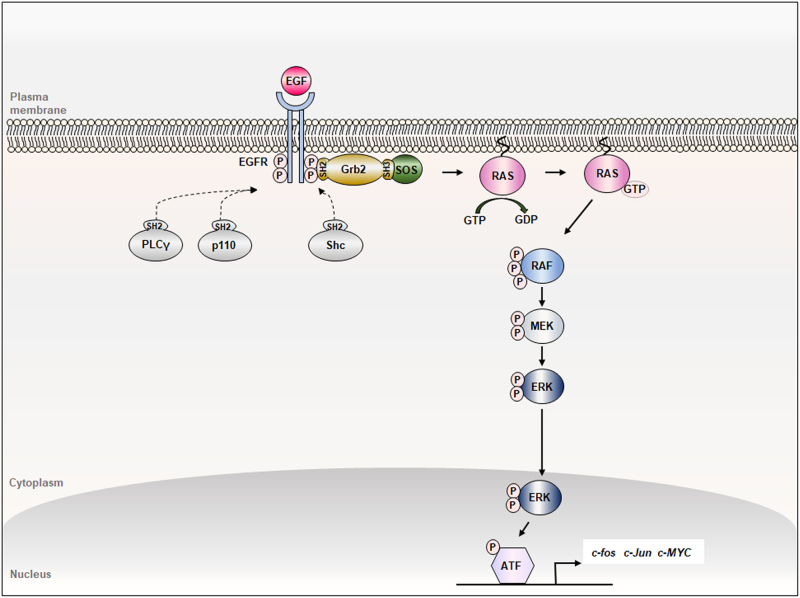
The RAS-ERK signaling pathway. Binding of EGF (epidermal growth factor) activates EGFR, providing the docking sites for several SH2 (src homology 2) domain-containing adaptor proteins such as Grb2 (growth factor receptor-bound protein 2), Shc (src homology 2 domain-containing), PLCγ (phospholipase C γ), and p110 subunit of the PI3 kinase (phosphatidylinositol-4,5-bisphosphate 3-kinase). As the major route involving the EGFR activation by EGF binding, Grb2-SOS (son of sevenless) complex is recruited to the receptor through SH2 domain, allowing SOS to activate RAS by exchanging GDP (guanosine diphosphate) to GTP (guanosine triphosphate). GTP-bound RAS activates the RAF (rapidly accelerated fibrosarcom)-MEK (MAPK/ERK kinase)-ERK (extracellular signal-regulated kinase) kinase cascade by series of phosphorylations. Phosphorlyated ERK translocates to nucleus and activates trasncription factors, such as ATF (activating transcription factor), leading to expression of the target genes

The RAS-ERK pathway mediates various cellular physiologies, including differentiation, survival, motility, and proliferation of cells.^20–23^ The signaling starts by binding a growth factor to the receptor tyrosine kinase (RTK), such as binding of epidermal growth factor (EGF) to the EGFR result in its dimerization and following autophosphorylation at the tyrosine amino acid residues provide docking sites for several SH2 domain-containing adaptor proteins, such as growth factor receptor bound protein 2 (Grb2), Shc, phospholipase C gamma (PLCγ), p110 catalytic subunit of PI3 kinase (Fig. 2).^24,25^ The Grb2 which present as a form bound to the guanine nucleotide exchange factor SOS with its SH3 domain, recruited to the active tyrosine phosphorylated EGFR via the SH2 motif. Activated SOS activates RAS family proteins via stimulating the exchange GDP- to GTP-bound form.^26–28^ Activated GTP-bound RAS proteins further activate the RAF-MEK-ERK kinase cascade by series of the phsphorylation events of the kinases. The active phosphorylated ERK translocate into the nucleus for activation of transcription factors like ATF through their phosphorylation. The activated transcription factors induce target genes, such as *c-MYC*, *c-fos*, and *c-Jun* involving regulation of various pathophysiological cellular processes including differentiation and transformation, and so on (Fig. 2).^29^

## The Wnt/β-catenin and RAS-ERK pathways interact in the colorectal tumorigenesis

**Fig. 3:**
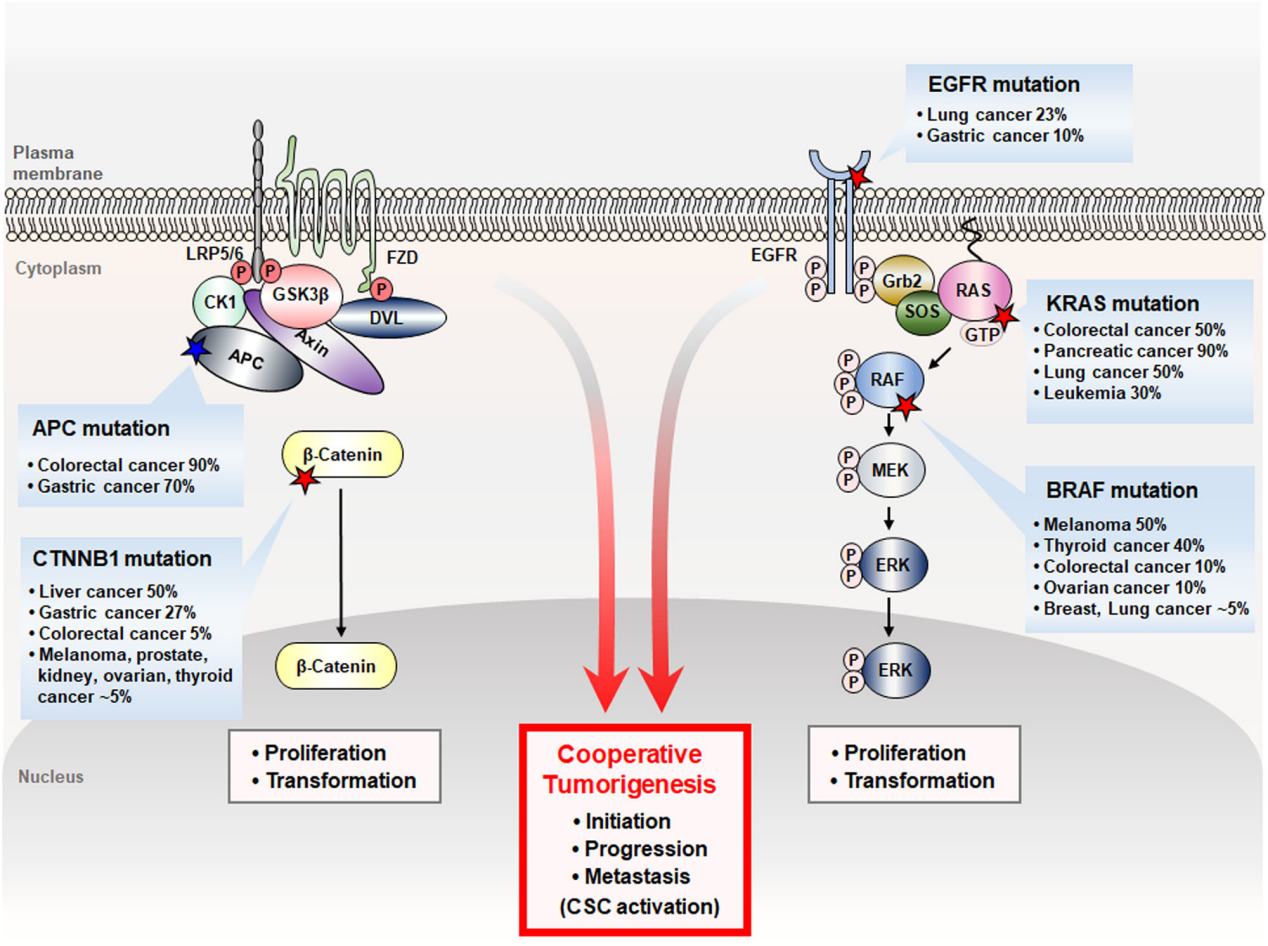
Aberrancies of the Wnt/β-catenin and RAS-ERK pathways and their cooperation in the tumorigenesis. Aberrant activations of the Wnt/β-catenin and RAS-ERK pathways caused by various mutations occur in various human cancers as described. When aberrancies of the two pathways occur together by mutations, such as *APC* and *KRAS*, they cooperate in the tumorigenesis and that results in the enhancements of the tumorigenesis at different stages of CRC (colorectal cancer), including initiation, progression, and metastasis, involving CSC (cancer stem cell) activation. Red star, gain-of-function mutation; Blue star, loss-of-function mutation

Due to essential roles in vital cellular processes, including the growth control, homeostasis, and regeneration of cells, the Wnt/β-catenin and RAS-ERK signaling pathways must be tightly regulated.^7,8,30,31^ Aberrant activations of each of these two pathways are known to play roles in various types of human cancers, including CRC (Fig. 3). Especially, in human CRC, *APC* mutations present as high as 90%, and *CTNNB1* mutations occur at a low frequency as 5% of the patients and these mutations have been observed to be mutually exclusive.^32^ *K-RAS* mutations occur as high as 50% of CRC patients, and those represent one of the most prevalent genetic alterations in CRC.^20^ Colorectal tumorigenesis often begins with precursor lesions in the colonic epithelium, and is driven by the accumulation of genetic and epigenetic alterations.^1^ The Wnt/β-catenin and RAS-ERK pathway gene mutations, such as *APC* and *KRAS* mutations occur in an order in CRC, and play roles in the initiation and progression of the tumorigenesis, respectively.^2^ Although the importance of the individual mutations of the Wnt/β-catenin and RAS-ERK pathway genes are well illustrated, their relationship to the tumorigenesis is poorly understood. The previous studies, however, indicated cooperation of the aberrant activation of two pathways in the pathogenesis induced by mutations of both pathway genes, such as by *Apc* and *KRas* or *CTNNB1* and *HRas* mutations in CRC and HCC, respectively.^3,4,33,34^ Although large cell dysplasia was observed in the hepatocytes, *HRas* mutation alone did not induce significant cancer phenotypes, but critical tumor development was observed when an additional mutation of *CTNNB1* was introduced into the HCC mice model.^4^ Both *APC* and *KRas* mutations not by their single-mutation critically enhanced both initiation and growth of colorectal adenocarcinoma,^3,34^ and even the tumor was progressed into the liver metastasis in a xenograft model used modified DLD-1 CRC cell line harboring both *APC* and *KRAS* mutations.^6^

Overall, colorectal tumorigenesis by both pathway gene mutations, such as *Apc* and *KRas* mutations attributed not only by effect of each pathway activation but also by cross-talk between the two pathways. These gene mutations cooperatively enhance various stages of the colorectal tumorigenesis, including initiation, progression, and metastasis, involving activation of CSCs (Fig. 3).

## A mechanism for the cross-talk between the Wnt/β-catenin and RAS-ERK pathways

**Fig. 4:**
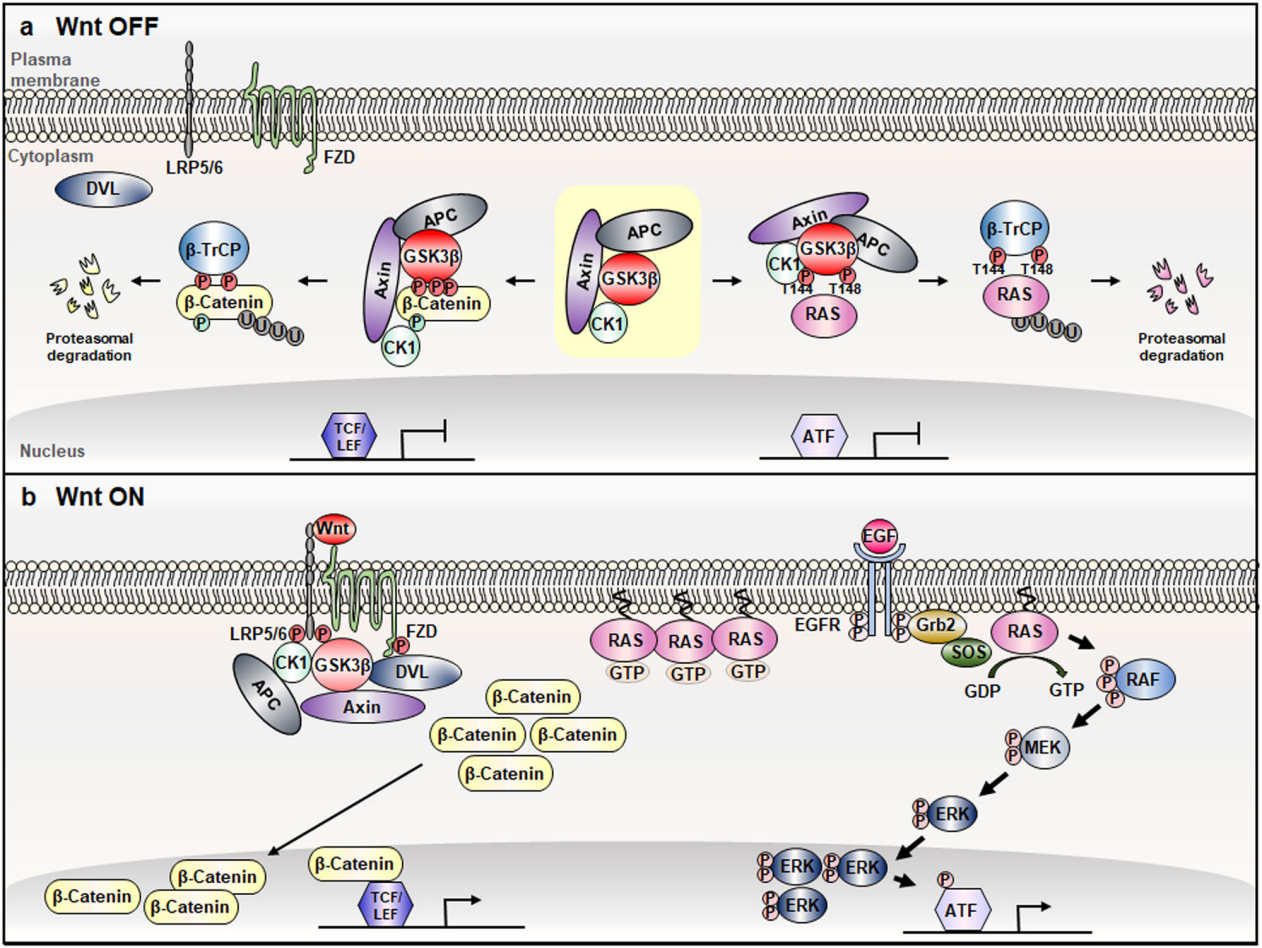
Cross-talk between the Wnt/β-catenin and RAS-ERK pathway. **a** In the absence of Wnt ligands, β-catenin is subjected to proteasomal degradation by association of the β-catenin destruction complex (yellow shaded) as described in Fig. 1a. The active GSK3β in the destruction complex can also phosphorylate RAS. The β-TrCP E3 ligase is recruited to both of the phosphorylated β-catenin and RAS proteins and subsequently degrades them via the polyubiquitination-dependent proteasomal degradation. Low β-catenin and RAS protein levels in cytoplasm ensure the repression of the signaling activities and target gene expressions. **b** In the presence of Wnt ligands, the dissociation of the destruction complex stabilizes both β-catenin and RAS in the cytoplasm, leading to the activation of the signaling pathways and target gene expressions

By series of studies, we previously identified regulation of the RAS-ERK pathway by the Wnt/β-catenin signaling, and that regulation directly related with the proliferation and transformation of cells.^35–38^ The Raf-1-MEK-ERK pathway immediately activated by stimulation with Wnt3a, revealed direct interaction of the two pathways.^38^ The Raf-1-MEK-ERK pathway regulation via the Wnt/β-catenin signaling and its roles in the proliferation and transformation of cells were further confirmed by the experiments modulating of the components of the Wnt/β-catenin signaling, such as Axin, APC, and GSK3β, and so on.^35–37,39^ The highlight in the cross-talk between the Wnt/β-catenin and RAS-ERK pathways is the stability regulation of RAS by the Wnt/β-catenin signaling involving the GSK3β kinase mediated phosphorylaton of RAS.^40^ In a resting status of Wnt/β-catenin signaling, HRas protein was subjected to the phosphorylations at the threonine (Thr)-144 and Thr-148 by GSK3β, and subsequently the β-transducin repeat-containing protein (β-TrCP) E3 linker is recruited to the phosphorylated Ras and degraded by polyubiqutination-dependent proteasomal degradation (Fig. 4a).^40^ The GSK3β phosphorylation sites, Thr-144 and Thr-148, are conserved in H, N, KRAS and, all these RAS isotypes are subjected to the regulation of protein stability by Wnt/β-catenin signaling.^6,40,41^ In a condition with Wnt stimuli, β-catenin is accumulated by escape from the degradation by dissociation of the β-catenin destruction complex (Fig. 1b). As β-catenin, RAS proteins are accumulated due to absence of the GSK3β-mediated their phosphorylations. The stabilized RAS proteins at plasma membrane activate the RAF-MEK-ERK cascade (Fig. 4b).

## Pathological significance for the stabilization of both β-catenin and RAS especially mutant KRAS by *APC* loss in the colorectal tumorigenesis

**Fig. 5:**
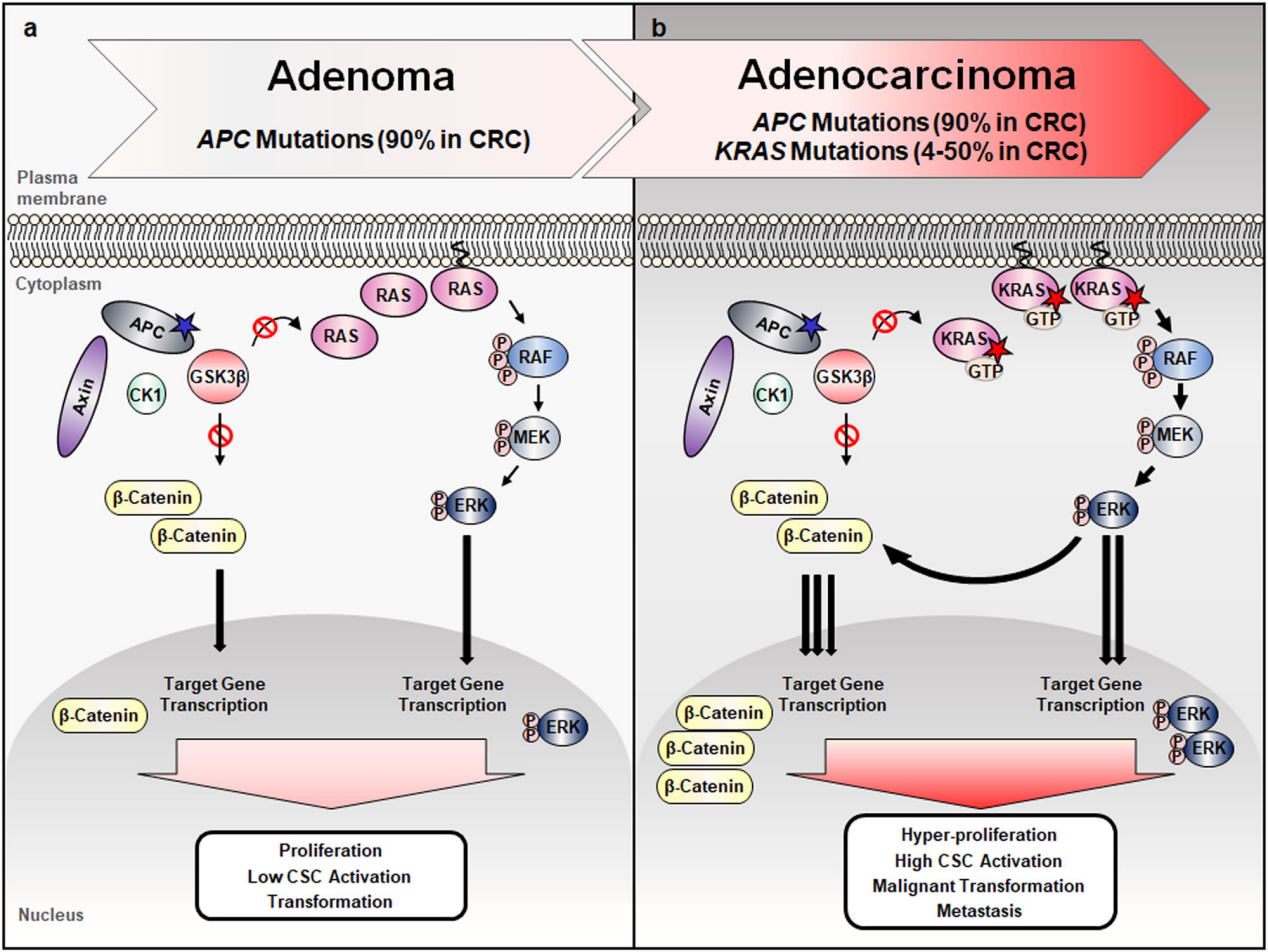
Pathological significance of the synergistic cooperation in the colorectal tumorigenesis by both *APC* and *KRAS* mutations and supporting mechanism. **a** Loss-of-function mutations of *APC*, observed in 90% of CRC patients, disrupt the destruction complex, lead to accumulation of β-catenin and RAS and thereby initiate adenoma formation. **b** When both *APC* and *KRAS* mutations occur, mutant KRAS proteins are accumulated by stabilization due to absence of its phosphorylation by inactivated GSK3β by *APC* loss. The Wnt/β-catenin signaling is strongly enhanced by its initial stabilization and further activation via the stabilized oncogenic mutant KRAS by *APC* loss. The additional activation of β-catenin signaling by the oncogenic KRAS occurs via a positive feedback loop through the MEK-ERK pathway, although in the detailed mechanism is not illustrated. The strong activation of the Wnt/β-catenin signaling involves malignant transformation accompanying the activation of CSCs

The *APC* and *KRAS* mutations occur as high as 90 and 40–50% of human CRC, and pathological significance of both β-catenin and RAS stabilization by *APC* loss is evidenced by co-increment of these proteins in human CRC patient tissues.^6,40^ There is a positive association between tumorigenesis and RAS stabilization induced by aberrant activation of Wnt/β-catenin signaling, such as by *APC* loss.^6,40^ Significance of the RAS stabilization especially mutant KRAS by hyper-activation of the Wnt/β-catenin signaling was evidenced by enhancement of both initiation and growth of tumors by both *APC* and *KRas* mutation. However, neither *APC* nor *KRAS* mutation alone induce severe CRC phenotype, especially *KRAS* mutation alone, in the mouse model.^6,40^ The highlight of outcomes by both *APC* and *KRAS* mutations in CRC is activation of CSCs and observation of liver metastasis in the xenograft mice model using a CRC cell line harboring both *APC* and *KRAS* mutation. The expression of CSC markers CD44, CD133, and CD166 were significantly induced by the two gene mutations although *KRas* mutation alone did not^6^ as previously reported that expression of a mutant *KRas* allele alone in mouse colonic cells failed to expand the stem cell population.^42^ Therefore, loss-of-*APC* is required for the mutant *KRAS*-induced CSC activation, and these results confirmed by critical enhancement of the spheroid forming ability of the CRC cells carrying both of the two gene mutations. In addition, both number and size of tumor formed by transplantation of the spheroids into flanks of the NOD/SCID mice were also increased 10-folds by simultaneous mutations of the two genes.^6^ The CSC activation by mutant KRas with *Apc* loss is attributed strongly enhanced activation of β-catenin by its initial activation and its further activation by the stabilized oncogenic KRas by *Apc* loss followed by activation of the downstream RAF-MEK and ERK cascade (Fig. 5). The β-catenin activation by KRas stabilization was inhibited together with suppression of the CSC markers by MEK inhibitor,^6^ indicates the secondary activation of β-catenin by stabilized KRAS is acquired by the MEK-ERK pathway although the in detailed molecular mechanism is not illustrated (Fig. 5). Overall, considering overexpression of both β-catenin and RAS especially mutant KRAS and their roles in the critical events of the tumorigenesis, inhibition of the Wnt/β-catenin and RAS-ERK signaling especially by destabilization of both β-catenin and RAS could be an ideal therapeutic strategy for treating CRC.

## Identification of compounds that destabilize both β-catenin and RAS via inhibiting the Wnt/β-catenin signaling

**Fig. 6:**
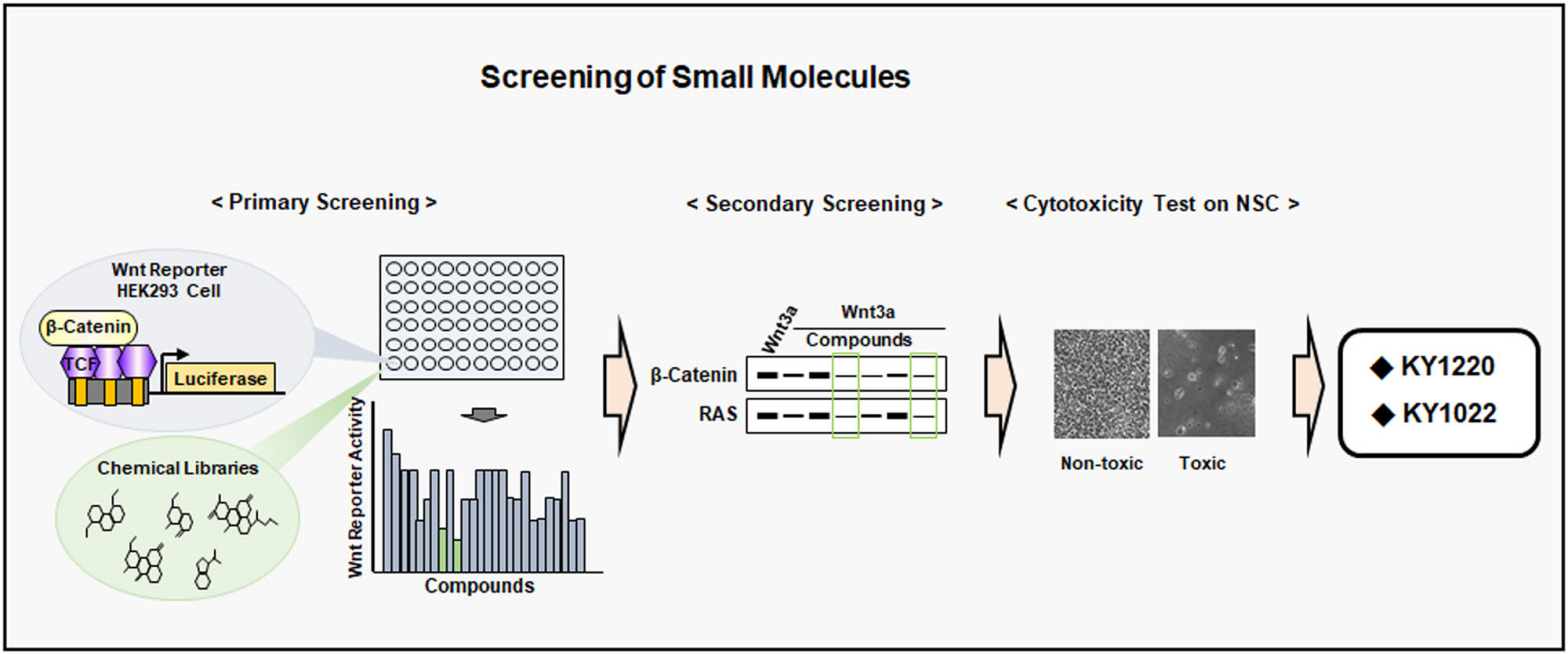
Screening procedures to obtain small molecules degrading both β-catenin and RAS. A schematic representation of screening procedures for identification of small molecules destabilizing both β-catenin and RAS. Through primary screening of chemical libraries for inhibitory effects on the Wnt reporter activities, the secondary screening for the effective reduction of both β-catenin and RAS protein levels by immunoblot analyses and following cytotoxicity tests by using NSCs (neural stem cells) to avoid potential toxic compounds, KY1220 and KY1022 were selected as representative compounds

To identify compounds destabilize both β-catenin and RAS via targeting the Wnt/β-catenin pathway, we initially screened a small molecule library by using HEK293 cells harboring the pTOPflash reporter which sensitively response to the β-catenin/TCF transcription factor in their chromosome.^41^ Small molecules inhibit the reporter activity stimulated by Wnt3a-conditioned medium (Wnt3a-CM) were selected. As a secondary screening, the compounds reduced both β-catenin and Ras levels were further screened by immunoblotting. Subsequently, potential toxic compounds were excluded from the candidates by using highly fragile neural stem cells for further characterization (Fig. 6). Through these series of procedures, we obtained several compounds for further characterization. KY1220 and KY1022, the representative candidate compounds, effectively inhibited the Wnt/β-catenin reporters with half-maximal inhibitory concentration (IC_50_) of 2.1 and 0.5 µM, respectively, destabilized both β-catenin and RAS, and were further characterized their functions.^41,43^ KY1220 and KY1022 inhibited both ERK and Akt pathways, and suppressed proliferation and transformation of CRC cells. Especially, these compounds effectively suppressed growth and transformation of various CRC cell lines which harboring either wild-type or mutant *KRAS* via degradation of both β-catenin and RAS via the enhancement of their polyubiquitination.^41,43^ KY1022 inhibited EMT, motility, invasion with induction of apoptosis of CRC cells harboring both *APC* and *KRAS* mutations.^43^ In addition, the small intestinal tumors of *Apc*^*Min/+*^*/KRas*^*G12D*^*LA2* mice were also suppressed by KY1220 and KY1022.^41,43^

## Production of KYA1797K, a functionally improved KY1220 mimetic, and its effectiveness on inhibition of tumors induced by both *Apc* and *Kras* mutations

**Fig. 7:**
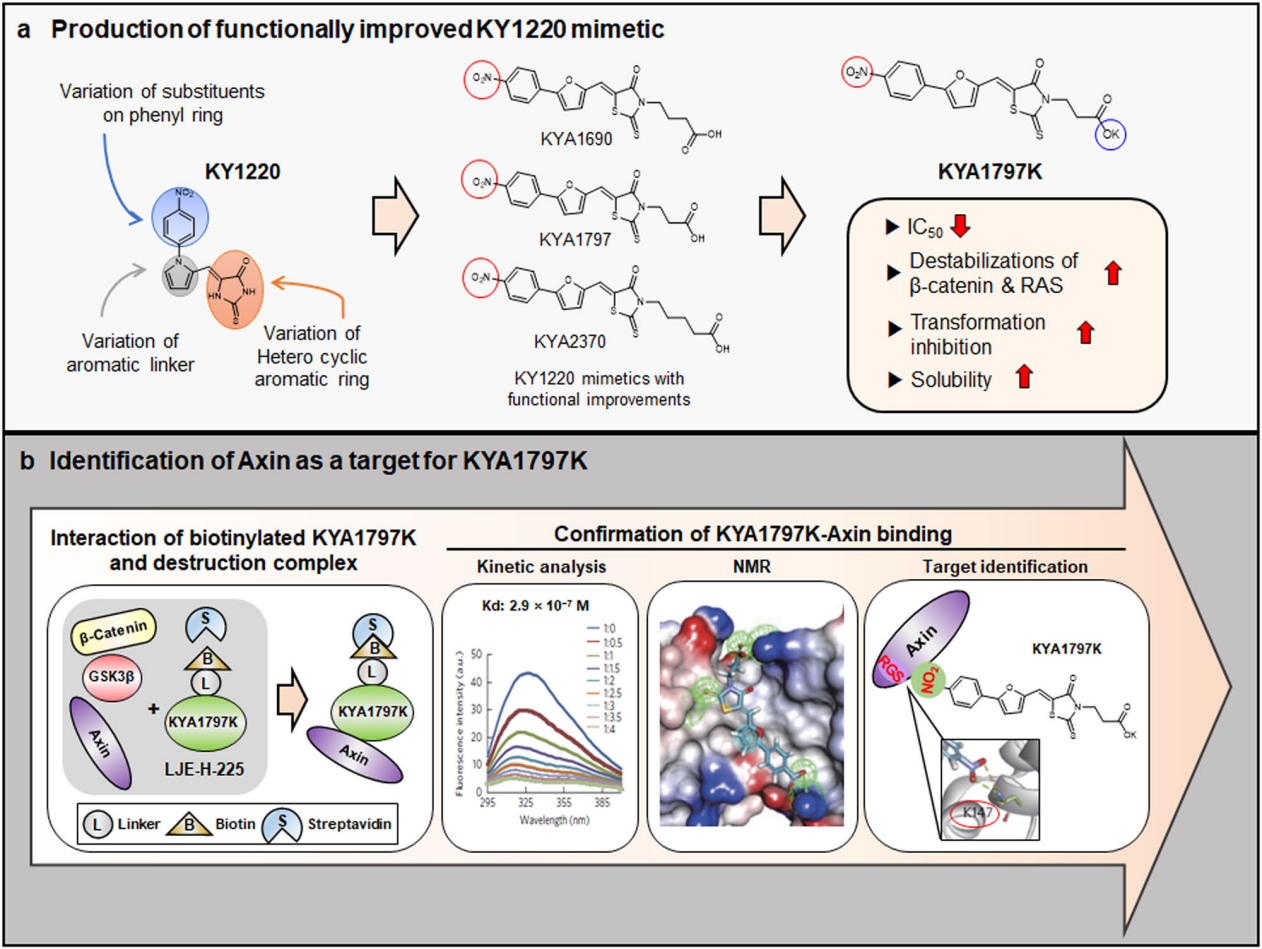
Production of KYA1797K, a functionally improved analog of KY1220, and identification of Axin as the target for KYA1797K. **a** Based on the structure of KY1220, a focused library was designed and selected three derivatives with NO_2_ moiety essential for the Wnt inhibitory effects (red circle) by improved inhibitory effect on the Wnt/β-catenin signaling. Among them, KYA1797K was produced as the most efficient analog with improved solubility (blue circle). **b** Among the candidate β-catenin complex proteins including Axin, GSK3β, β-catenin, Axin was specifically pulled-down with the KYA1797K attached biotinylated active compound LJE-H-225. Binding of Axin-RGS (regulator of G-protein signaling domain of Axin) with KYA1797K was analyzed with a dissociation constant (*K*_d_) of 2.9 × 10^−7^ M. Further confirmation of KYA1797K interaction with Axin-RGS by NMR (nuclear magnetic resonance) spectra specified direct interaction of K147 with the NO_2_ moiety of the KYA1797K. Reproduced with permission and adapted from: Cha, P.H. et al.^41^

To generate more effective compounds, we synthesized derivatives of KY1220 by chemical synthesis, and selected several compounds improved their capabilities to degrade both β-catenin and RAS and to inhibits transformation of SW480 cells with both *APC* and *KRAS* mutations.^41^ All the compounds improved their functions has NO_2_ moiety at the phenyl ring (Fig. 7a). KYA1797K, one of the KY1220 mimetics which improved in its capabilities in both β-catenin and RAS degradations with the IC_50_ value of 0.75 µM in the inhibition of the TOPflash reporter, have phenyl ring with the NO_2_ moiety essential for functionality, aromatic linker, and heteroaromatic ring, and potassium salt to improve solubility of the compound (Fig. 7a). Compared to KY1220, KYA1797K more effectively suppressed colony formation of CRC cells.^41^ KYA1797K specifically inhibited the Wnt/β-catenin and RAS-ERK pathways as shown by specific suppressions of the pTOPflash and MAPK/ERK reporters, but not the Notch, p53/DNA damage, TGFβ, cell cyle/pRb-E2F, NF-kB, Myc/Max, Hypoxia, and MAPK/JNK pathway reporters.^41^ The role of KYA1797K in the suppression of tumor was confirmed by inhibition of the tumor growth in mouse xenografts of D-MT cells, the DLD-1 derivative CRC cell line expressing *APC* and *KRAS* mutations.^41^ The tumor inhibitory effect of KYA1797K was further confirmed by suppression of both initiation and progressive growth of small intestinal tumors generated by hybrid *Apc*^Min/+^ and *KRas*^*G12D*^*LA2* compound mice.^41^

## KYA1797K directly interacts at the Axin-RGS domain, and induce degradations of both β-catenin and RAS via activation of GSK3β followed by their phosphorylation

The β-catenin destruction complex as the target of KYA1797K was initially predicted by measuring the differences in the effects of this compound on the phosphorylation status of the signaling components β-catenin, GSK3β etc. and on the binding affinities between the proteins in the “β-catenin destruction complex”.^41^ Axin was identified as a specific target of the KYA1797K by pull-down of Axin with the active biotinylated compound (LJE-H-225), not with the inactive control biotinylated compound (LJE-H-274) following confirmation of the specific interaction of LJE-H-225 with recombinant Axin (Fig. 7b).^41^ The kinetic analysis using the fluorescence spectroscopy determined a dissociation constant (*K*_d_) for KY1797K-Axin binding as 2.9 × 10^−7^ M. The regulator of G-protein signaling (RGS) domain of Axin (RGS-Axin) was characterized as a site of KYA1797K interaction by analyzing the RGS domain deleted Axin mutant followed by abolishment of its RAS degrading ability. We further predicted several amino acid residues involving KYA1797K by chemical shift perturbations using NMR spectra and confirmed lysinen-147 (K147) as a most important residue in the function by KYA1797K.^41^ This K147 in the RGS domain of Axin is essential for KYA1797K binding, and it directly forms hydrogen bond with the NO_2_ at the phenyl ring (Fig. 7b).

## KYA1797K is a potential drug candidate suppressing CRC resistant to cetuximab, the humanized antibody drug targeting EGFR

**Fig. 8:**
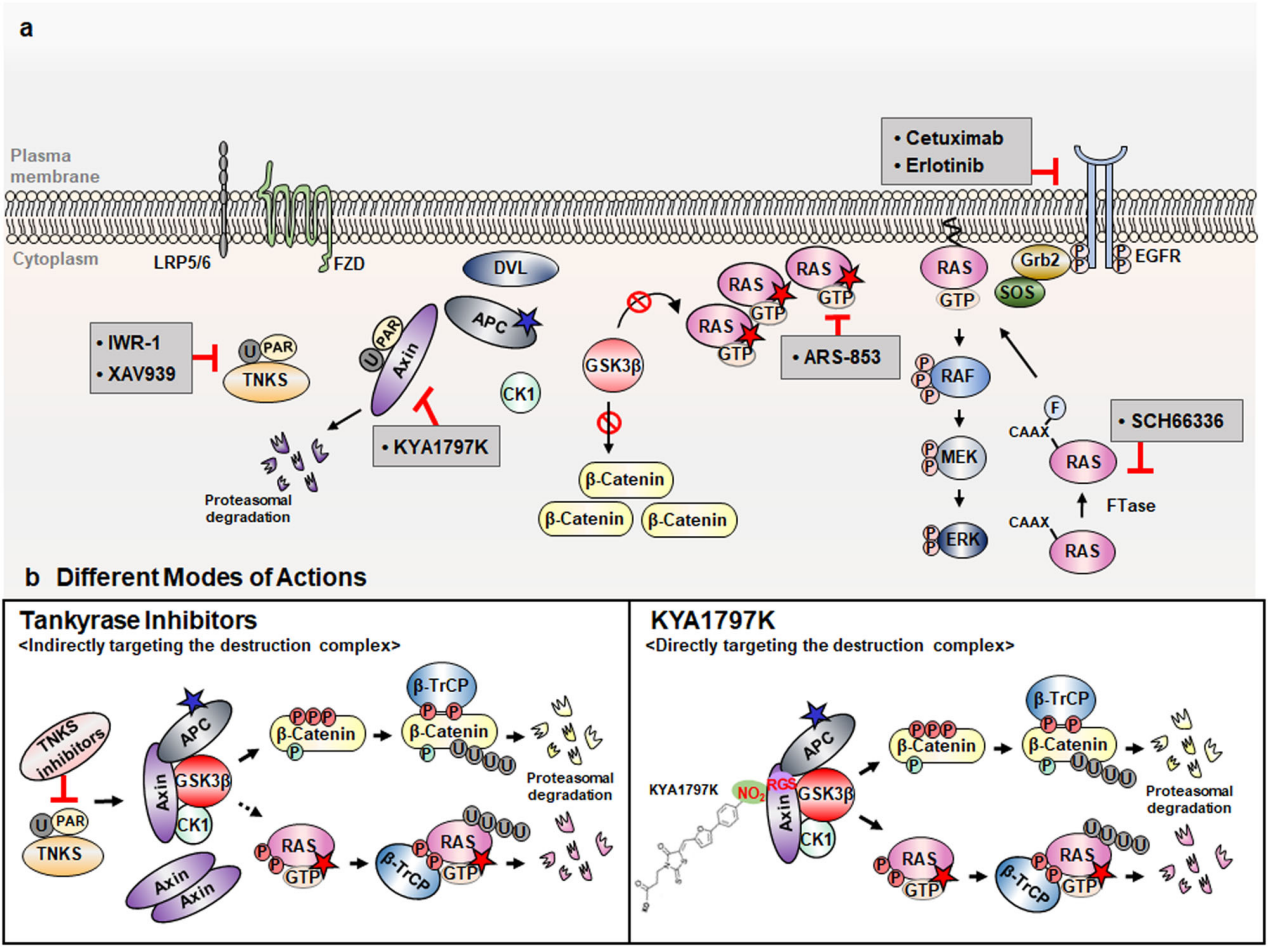
KYA1797K as a potential strategy to overcome resistance to EGFR targeting drugs in CRC. **a** A schematic representation of several therapeutic agents targeting the EGFR-RAS-ERK pathway including EGFR antibody drug cetuximab, FTase (farnesyl transferase) inhibitor SCH66336, KRAS-G12C specific inhibitor ARS-853 and those targeting Wnt/β-catenin pathways, including the IWR-1 and XAV939 TNKS (tankyrase) inhibitors and KYA1797K targeting RGS-Axin. **b** Differences in the mode of actions of TNKS inhibitors and KYA1797K. Left: TNKS inhibitors indirectly target the destruction complex by inhibiting the TNKS-mediated Axin destabilization which may change the balance of the β-catenin destruction complex components. Right: KYA1797K directly targets the destruction complex by binding to Axin-RGS which enhances the formation of the β-catenin destruction complex leading to increased GSK3β activity without changing the stoichiometry of the β-catenin destruction complex. F, farnesylation; PAR, poly-ADP-ribosylation; Dotted line, not experimentally confirmed

The RAS-ERK pathway targeting drugs especially those targeting RAS have been intensively investigated several decades as major subjects of the anti-cancer drug development.^44,45^ Despite the effort to develop drugs controlling RAS such as small molecules inactivating Ras by direct binding and by inhibiting farnesyltransferases (FTase) mediated membranous localization and alternative approaches, such as synthetic lethality and small interfering RNA mediated reduction of Ras, no effective pharmacologic inhibitors of the Ras have reached the clinic, prompting the widely held perception that RAS proteins are “undruggable”.^46–53^ As one of the continuous efforts to develop small molecules control oncogenic RAS, ARS-853 (Fig. 8a), a selective and covalent inhibitor of KRAS G12C, has been developing as a candidate for anti-cancer drug inhibits G12C mutant KRAS, but this small molecules limited in general usage.^54–56^

Here we introduce a small molecular approach degrading both β-catenin and RAS, such as usage of the KYA1797K and mimetics as candidates controlling RAS activity by reducing its level (Fig. 8a).^41,43^ KYA1797K which binds the RGS domain of Axin suppress CRC induced by both *KRAS* and EGFR abnormalities, including both their mutations and overexpression which occur most of human CRC patients due to the *APC* mutations.^6,40^ The preceding experiments show that KYA1797K degraded both wild-type and mutant Ras proteins and successfully suppressed the transformation of CRC cells, as well as the progression of tumors^41^ which are insensitive to the cetuximab, the antibody based EGFR therapy, attributed by *KRAS* mutations (Fig. 8a).^57^ Tankyrase (TNKS) inhibitors, such as IWR-1 and WAV939 are known to suppress Wnt/β-catenin signaling pathway through Axin stabilization via inhibition of TNKS-mediated poly-ADP-ribosylation (PARsylation) (Fig. 8a).^58,59^ TNKS inhibitors reduce both β-catenin and RAS levels without dose-dependence (Fig. 8b).^41^ Differently with TNKS inhibitors functioning via chancing stoichiometry of the β-catenin complex proteins, KYA1797K directly targets the β-catenin destruction complex by binding to Axin-RGS domain and dose-dependently reduces both β-catenin and Ras levels via changing status of the complex without changing levels of the component proteins (Fig. 8b). KYA1797K degrades H, NRAS, as well as KRAS, which have the conserved GSK3β phosphorylation sites, and both wild-type and mutant RAS proteins. Considering the results that wild-type H and NRAS is required for the mutant KRAS-induced tumor progression,^60^ degradation of wild-type H and NRAS together with the mutant KRAS provide a benefit for the application of KYA1797K and derivatives as anti-cancer drug candidates.

## Conclusion and future perspectives

The genetics of multistep colorectal carcinogenesis has been extensively studied over the past decades, and frequent alterations of Wnt/β-catenin and RAS/ERK pathway genes in different cancers especially human CRC has been recognized. In addition, cooperative interactions between mutations of the Wnt/β-catenin and Ras-ERK pathway genes have been shown by various genetic studies mostly using the mouse system. The most frequent mutations in the two pathway genes involving their interaction in the tumorigenesis is the *APC* and *KRAS* mutations in CRC. Pathological significance of these two mutations was evidenced by correlative increment of both β-catenin and RAS in tumor tissues of CRC patients harboring an *APC* mutation in most cases. On the base of these results together with identification of the detailed mechanism of common stability regulations of both β-catenin and RAS lead us to develop small molecules degrading both β-catenin and RAS protein. KYA1797K, a representative such compounds illustrated its mode of action binding the RGS domain of Axin for GSK3β activation, suppressed the growth of various CRCs, including those resistant to the EGFR targeting therapies by *KRAS* mutations.

Overall, the idea for the simultaneous destabilization of β-catenin and RAS via targeting Wnt/β-catenin pathway may serve as an effective anti-cancer strategy for CRCs and other cancer types attributed not only by *APC* or *KRAS* mutations but also by level increments of both β-catenin and RAS. However, considering the small molecular approach degrading β-catenin and RAS is not effective in cancer cells with non-degradable β-catenin mutation,^41^ for the best outcome, the strategy should be carefully applied based on the individual’s genetics following precision oncology concept.^61^
